# Chinese Proprietary Herbal Medicine Listed in ‘China National Essential Drug List’ for Common Cold: A Systematic Literature Review

**DOI:** 10.1371/journal.pone.0110560

**Published:** 2014-10-20

**Authors:** Wei Chen, George Lewith, Li-qiong Wang, Jun Ren, Wen-jing Xiong, Fang Lu, Jian-ping Liu

**Affiliations:** 1 Centre for Evidence-Based Chinese Medicine, Beijing University of Chinese Medicine, Beijing, China; 2 Primary care and population Sciences, Medical School, University of Southampton, Southampton, United Kingdom; Central South University, China

## Abstract

**Objective:**

Chinese proprietary herbal medicines (CPHMs) have long history in China for the treatment of common cold, and lots of them have been listed in the ‘China national essential drug list’ by the Chinese Ministry of Health. The aim of this review is to provide a well-round clinical evidence assessment on the potential benefits and harms of CPHMs for common cold based on a systematic literature search to justify their clinical use and recommendation.

**Methods:**

We searched CENTRAL, MEDLINE, EMBASE, SinoMed, CNKI, VIP, China Important Conference Papers Database, China Dissertation Database, and online clinical trial registry websites from their inception to 31 March 2013 for clinical studies of CPHMs listed in the ‘China national essential drug list’ for common cold. There was no restriction on study design.

**Results:**

A total of 33 CPHMs were listed in ‘China national essential drug list 2012’ for the treatment of common cold but only 7 had supportive clinical evidences. A total of 6 randomised controlled trials (RCTs) and 7 case series (CSs) were included; no other study design was identified. All studies were conducted in China and published in Chinese between 1995 and 2012. All included studies had poor study design and methodological quality, and were graded as very low quality.

**Conclusions:**

The use of CPHMs for common cold is not supported by robust evidence. Further rigorous well designed placebo-controlled, randomized trials are needed to substantiate the clinical claims made for CPHMs.

## Introduction

Common cold is often caused by rhinovirus [1]. Common symptoms include cough, sore throat, runny nose, fever, and etc. It is one of the most widespread illnesses in the world. On average, adults have two to three infections a year [2] and children have six to twelve a year [3]. The common cold is a mild and self-limiting illness that almost always resolves spontaneously. To date there is no effective treatment for common cold and the routine use of antibiotics for the common cold is not recommended [4]. Some alternative treatments are used for common cold; however, there is insufficient scientific evidence to support their use [3]. The recommended first line treatment for common cold is usually medication for symptom control to avoid the unnecessary prescription of antibiotics and the consequent risk of adverse drug reactions and antimicrobial resistance.

Chinese herbal medicines have long history in China for the treatment of common cold. It was recorded more than 2000 years ago in ‘Inner Canon of Huangdi’, the most acknowledged classics of TCM, that ‘pathogenic wind can cause cold’ [5]. Traditional Chinese medicine (TCM) practitioners and general public have deep belief that herbal medicines are effective in alleviating symptoms and shortening the duration of the common cold. Chinese proprietary herbal medicine (CPHMs) is an important component of Chinese herbs. It refers to Chinese herbs that mainly produced by modern manufacturing methods. CPHMs include different formulations such as powder, granule, pastille, tablet, and capsule, and are widely accepted by the Chinese population due to the convenience of application. Until now, More than two hundreds CPHMs have been authorized and listed in the ‘China national essential drug list’ (EDL), which is approved by the Chinese Ministry of Public Health and is regarded as the accepted reference point for the medicines used in medical institutions in China.

The World Health Organization (WHO) strategy calls for evidence-based TCM. In order to ensure evidence-based practice, we conducted systematic evaluation of all the CPHMs listed in the EDL 2012 for the treatment of common cold. Although RCT was acknowledged as the gold standard for therapeutic evaluation, we didn’t want to neglect other designs because they might accounts for a large part of the clinical evidence. Our aim was to provide a well-round clinical evidence assessment of CPHMs for common cold based on a systematic literature search.

## Methods

### Inclusion criteria

Children and adults with the common cold were included. Typical symptoms include cough, sore throat, runny nose, sneezing, fever, and etc. Colds caused by influenza, acute bronchitis developing from a case of common cold, upper respiratory tract infection caused by bacteria, and patients concurrently suffering from other infectious or febrile diseases were excluded. There was no restriction on age and sex.

The interventions were confined to CPHMs listed in EDL 2012 for common cold. There was no restriction on study design. Systematic reviews (SR), randomized controlled trial (RCT), quasi-randomized controlled trial (Q-RCT), non-randomized controlled trial (NRCT), controlled before-and-after study (CBA), prospective cohort study (PCS), retrospective cohort study (RCS), historically controlled trial (HCT), nested case-control study (NCC), case-control study (CC), cross-sectional study (XS), before-and-after comparison (BA), case reports (CR), and case series (CS) were all identified. In order to reduce misclassification and inconsistencies, we used explicit study design features (as shown in Table S1) to facilitate our judgement on study design. Before assessment, two evaluators (WC, LQW) were trained to apply these standards consistently. Disagreement was resolved by discussion and consensus was reached through a third party (JPL).

For RCT, Q-RCT, NRCT, CBA, PCS, RCS, HCT, NCC, and CC studies, CPHMs compared with no treatment, placebo or conventional medication were included. Studies compared CPHM plus other interventions with other interventions alone were also included. CPHM combined with other TCM therapies (including acupuncture) compared with non-TCM therapies were excluded. Studies that compared different CPHMs were excluded. There was no restriction on language and publication type. Literatures that reported same data were be regarded as multiple publications and excluded.

### Search strategy and study selection

The CENTRAL (2012, Issue 12) (http://www.cochrane.org/editorial-and-publishing-policy-resource/cochrane-central-register-controlled-trials-central), MEDLINE (http://www.ncbi.nlm.nih.gov/pubmed/), EMBASE (http://www.elsevier.com/online-tools/embase), SinoMed (Chinese Biomedical Literature Service System) (http://www.sinomed.ac.cn/), Chinese VIP information (VIP) (http://www.cqvip.com/), Chinese National Knowledge Infrastructure (CNKI) (http://www.cnki.net/),, China Important Conference Papers Database (http://www.cnki.net/), and China Dissertation Database (http://www.cnki.net/) were searched from their inception to 31 March 2013. The following search terms were used individually or combined: ‘cold’, ‘nasopharyngitis’, ‘acute viral rhinopharyngitis’, ‘acute coryza’, ‘shang feng (cold in Chinese)’, ‘wai gan (cold in Chinese)’, and ‘feng han (cold in Chinese)’.

Website of the clinical trials registry including Chinese clinical trial registry (http://www.chictr.org/) and international clinical trial registry by U.S. national institutes of health (http://clinicaltrials.gov/) were also searched for ongoing registered clinical trials.

Two authors conducted the literature search (LQW, JR), study selection (WJX, FL) and data extraction (WC, LQW) independently. We extracted authors’ name and title of study, year of publication, study design (detail of randomization if the study was RCT), sample size, demographic characteristics of the participants, name and component of CPHM, treatment process, detail of the control interventions, outcome and adverse effect for each study. Double check was conducted to reduce inconsistencies. Disagreement was resolved by discussion or through a third party (JPL).

### Quality assessment

Two authors (WC, LQW) evaluated the quality of included studies independently. The quality of included RCTs were assessed by using the risk of bias tool according to the ‘Cochrane Handbook of Systematic Reviews of Interventions’ (Chapter 8) to address the following five criteria: random sequence generation, allocation concealment, blinding of participants and personnel, blinding of outcome assessment, incomplete outcome data, and selective reporting [6]. The quality of all the included trials was categorized to low/unclear/high risk of bias.

For the other study designs, different criteria were used. AMSTAR (A Measurement Tool to Access Reviews) [7] were used to assess the quality of SR, MINORS (Methodological Index for Non-Randomized Studies) [8] was used for NRCT, and NOS (Newcastle-Ottawa Scale) [9] was used for cohort and case-control studies. Due to the lack of acknowledged tool or scale for CR and CS, the quality assessment of these two studies was based on the explicit diagnostic criteria, detailed description of demographic characteristics, intervention, and acknowledged outcome measurements.

### Data analysis

SPSS 19.0 statistics software (480c9826941a904069d8) was used for data analyses. Data were summarized using relative risk (RR) with 95% confidence intervals (CI) for binary outcomes or mean difference (MD) with 95% CI for continuous outcomes.

## Results

### Description of studies

**Figure 1:**
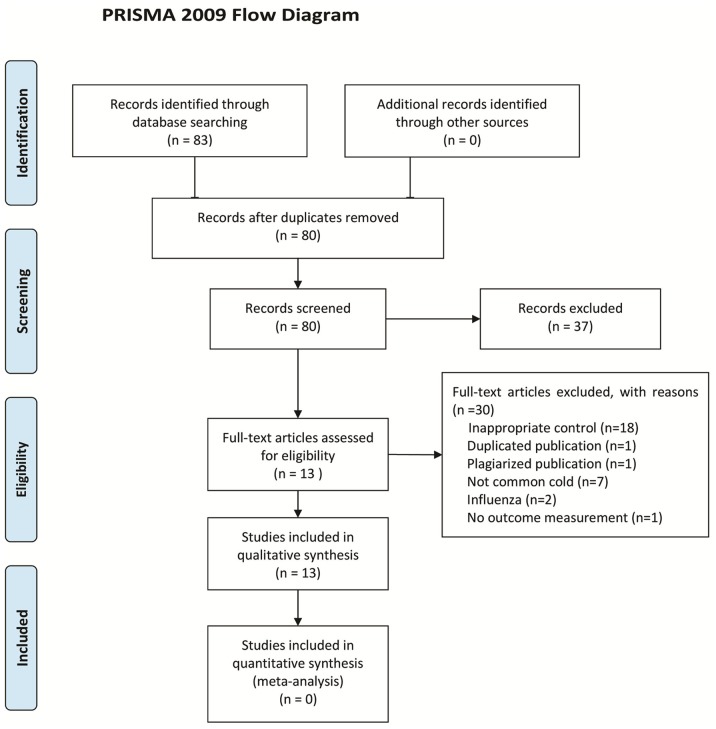
PRISMA 2009 Flow Diagram.

Thirty-three CPHMs were listed in EDL 2012 for the treatment of common cold (the compositions, indications, and dosage of the 33 CPHMs were shown in Table S2). After primary searches, 83 citations were identified. The majority was excluded due to obvious ineligibility (for example, animal experiment, influenza, bacterial infection of the upper respiratory tract, and etc), 46 full text papers were retrieved and 13 studies [10]–[22] fulfilled the inclusion criteria (Figure 1). The excluded studies and reason for exclusion were listed in Table S3.

Search results showed that only 7 CPHMs had published supporting clinical evidence. These CPHMs were Chaihu injection (5 CSs, 1 RCT), Qingre Jiedu granules (1 CS, 1 RCT), Huoxiang Zhengqi liquid (1 CS), Ganmao Qingre granules (1 RCT), Shuanghuanglian oral liquid (1 RCT), Xiaoer Baotaikang granules 1 RCT), and Xiaoer Resuqing oral liquid (1 RCT). A total of 6 RCTs and 7 CSs were included. No other study design was identified. All the studies were conducted in China, and published in Chinese between 1995 and 2012. The first RCT was published in 1997 [21] and the rest were all published after 2007. No studies report on informed consent or on whether they were properly approved by an IRB. Only one trial [10] revealed funding sources (Taizhou Municipal Science and Technology Project).

The characteristics of included studies were listed in Table S4. A total of 2643 participants with common cold were involved, ranging from 20 to 1560 per study. Eight studies included children [10], [11], [13], [16], [19], [20], [22], 1 study included adults [17], and 4 studies included both children and adults [12], [14], [15], [18]. Five studies provided information on patients’ syndrome differentiation (*Bianzheng*, TCM diagnosis) [16]–[19], [21]. One trial used the diagnostic criterion for common cold of textbook ‘practical paidonosology’ [10], 1 trial used the diagnostic criterion of the China State Administration of Traditional Chinese Medicine, and the other 6 studies did not report their diagnostic criteria [11]–[15], [20].

The routes of administration for CPHMs were quite diverse including oral intake, acupuncture point injection, intramuscular injection, retention enema, and nasal dripping. The treatment period was 3 to 5 days [11], [14]–[17], [19], [21], [22]. Four studies did not provide treatment period [12], [13], [18], [20]. The reported outcomes were duration of fever, body temperature, or clinical symptoms improvement rate within a particular time. Clinical symptoms improvement rate was a composite measurement including a number of symptoms, of which body temperature was the vital one. Adverse events were reported in only 2 studies [12], [18]. In Li 1997 [12], no allergic reaction was found. In Jiang 2012 [18], skin flushes, dizziness, and bitter taste in mouth were reported. While in the other 11 studies, the researchers did not reported whether or not they had monitored adverse events.

### Therapeutic effects

Because common cold was a mild and self-limiting illness, we could not discriminate its spontaneous recovery with therapeutic effects by using the design of CSs. Therefore, we only calculated the therapeutic effects of CPHMs of RCTs (shown in Table S5). Results showed that Chaihu injection given at acupuncture point (LI 11) could shorten fever duration, Qingre Jiedu granules, Shuanghuanglian oral liquid, and Xiaoer Baotaikang granules had better clinical symptoms improvement rate within 3 days, and that Xiaoer Resuqing oral liquid had better clinical symptoms improvement rate within 5 days.

### Methodological quality

Most of the included RCTs were of general poor methodological quality according to the predefined quality assessment criteria. Although ‘random allocation’ was mentioned in all RCTs, no trial described the methods for random sequence generation. We could not judge whether or not it was conducted properly due to the insufficient information. Allocation concealment and blinding were not reported in any RCT. No trial reported drop-outs or mentioned intention-to-treat analysis. Selective reporting was generally unclear in the RCTs due to the fact that all the RCT did not register before their start and we could not access to their trial protocol.

The included CSs were of very low quality. Most of the CSs did not reported diagnostic criterion, therefore it was possible that patients of influenza or upper respiratory tract infection caused by bacteria were included. Most of CSs did not provide a detailed description of baseline characteristics or treatment strategy. Moreover, most CSs used subjective outcome measurement such as cure rate, which was a self defined criteria and lacked unambiguous definition on outcome. Therefore made it difficult to interpret the effects even if the reported result was positive.

## Discussion

In this review, only 7 CPHMs listed in EDL 2012 had clinical evidence to support their use for the common cold, and most of them only had one RCT or CS as supporting evidence. In addition, the evidence quality level of these 7 CPHMs were low due to the limitations in the design and implementation of studies. All studies had a very high likelihood of bias. Therefore the therapeutic effect of CPHM for the common cold should be taken in caution. More importantly, the fact that more than 80% of the CPHMs recommended in the EDL 2012 were not supported by clinical evidence revealed the enormous lack of evidence base that currently underpinned clinical use and policy making in China.

In August 2009, the ‘Chinese national essential drug system’ was officially launched and implemented. The ‘Chinese national essential drug list’ (2009 edition) has been issued in the same year [23]. The EDL was adjusted every 3 years and in the year 2012, the newest 2012 edition was published. The EDL 2012 contains 520 medicines, including 317 chemicals and biological products and 203 CPHMs. The CPHMs listed in EDL cover 137 kinds of internal medicine, 11 kinds of surgical medicine, 20 kinds of gynecological medicine, 7 kinds of ophthalmological medicine, 13 kinds of otorhinolaryngological medicine, and 15 kinds of orthopedics and traumatological medicine. The scope and number of CPHMs in the EDL 2012 was listed in Appendix S1. More than 3,100 medical and clinical experts had been assembled to evaluate the safety, effectiveness and economy of CPHMs. The selection process of medicine into EDL was strictly in accordance with the principle that they ‘must be preventive and curative, safe and effective, affordable, easy to use, think highly of both Chinese and Western medicine’ [24]. The detailed procedure for evaluation was not available because they were confidential files. However, our study demonstrated that they were less likely to be ‘evidence-based’ and revealed the sharp contrast between the policy and priority given to by the Chinese government to TCM.

In our review, the control interventions included antibiotics, antivirus drugs, and antipyretic and analgesic drugs. It was known that antibiotics had no effect against viral infections. Ribavirin and moroxydine were not recommended treatments for common cold. For mild and self-limiting illness which had no proved effective treatment, like common cold, randomized placebo controlled trial was the best study design to investigate the therapeutic effect. However, in this review, we did not find placebo controlled RCT. In addition, all the clinical studies were of poor methodology, which frequently happened for Chinese clinical studies. For RCTs, methodology such as randomisation, blinding and placebo controls were not used. No trial reported drop-out or withdrawal, or mentioned intention-to-treat analysis. Poor methodology suggested the positive interpretation of the therapeutic effect of CPHMs could be biased, so claims about their effectiveness should be interpreted with caution. For CSs, we could not discriminate spontaneous recovery with therapeutic effects using the design of CSs due to the lack of comparison. The positive result itself drawn from a CS was not reliable. In addition, all the included CSs had poor methodology quality which was embodied in lack of diagnostic criterion, inadequate description of baseline characteristics or treatment strategy, and inappropriate outcome measurement, and therefore downgraded the reliability of the positive results. We thought part of the reason for the poor quality of TCM studies was that the research training in evidence-based medicine and critical appraisal had only just begun in China. There remains urgent need to train Chinese researchers in conducting unbiased trials in the future. Recently the government had increased the investment in TCM research, and many research activities were on-going in academic institutions and universities. With increasing awareness of the international guidelines for reporting of clinical trials, we hope the picture may change in future.

In the era of EBM, we need better evidence for CPHMs, and to achieve this we need to do thoughtful, placebo-controlled trials with proper randomization and blinding and, above all else, we need to think carefully about inappropriate control and outcome assessment. In the literature searching, a total of 18 trials were excluded due to inappropriate control. As a control, the drug should be definite effective or definite ineffective, or was widely used in clinical practice. During the literature searching, we found lots of trials which compared CPHM with another Chinese herb. We could not prove that the Chinese herb used as control was effective for common cold because we could not find any previous placebo-controlled trials on the treatment of this Chinese herb. Therefore, we have to exclude them. Using two different Chinese herbs as mutual comparison was a common mistake in TCM clinical trials in China and future researchers should be aware of this and make the correction. In our review, all the Chinese studies were so dependent on the presence or absence of fever as opposed to other symptoms, and this was at odds with the way Western medicine tends to design its evaluation of upper and lower respiratory tract infections. Most of the studies used body temperature or duration of fever as the main or exclusive outcome. For the studies that used composite criteria such as ‘clinical symptoms improvement rate’, body temperature was also a vital component. However, fever is just one of the symptoms and by no means the primary outcome because simple respiratory infections caused a range of symptoms (sore throat, muscle aches, sometimes gastrointestinal upset, runny nose, etc). Our suggestion to the future researchers was to use well validated outcome measurements and to consider all the related symptoms. A patient-reported daily symptom diary including symptom variables giving each symptom a score would be a valuable approach. Patients could also completed Likert scales of how satisfied or concerned they were with different aspects of treatment. These Likert scales have previously been shown to be reliable, have good construct validity, and predict illness duration [25]–[27].

In our review, we searched all studies assessing the CPHMs for treatment of common cold regardless of study design. However, only CSs and RCTs were identified. The possible reasons might be that in China few TCM practitioners, researchers, and journal editors have received scientific research methodology training [28]–[30]. Articles based on case series were more acceptable during the l990s and early 2000’s as they were closer to clinical practice. Since the year of 2000, with the spread of evidence-based medicine, more and more TCM practitioners began to use RCTs because it was regarded as the gold standard for evaluating therapeutic effects. That was why we did not find other study designs.

In our review, all the included 13 studies were published in 12 different journals. The 12 journals were all legitimate Chinese medical journals. However, only 5 journals were rated as the core journals in China. The grade of journal was thought related to the quality of the trials it published. We suggest the editors of all TCM journals to be trained on clinical research methodology to ensure the scientific scrutiny for published researches.

The report of adverse events of CPHMs was not adequate. One study [12] reported that no allergic reaction was found; one study [18] reported skin flushes, dizzy and bitter taste. However, their reports were too brief to providing useful information. Turner observed that adverse events were often not well-reported in CAM RCTs [31]. In China, it was commonly believed that CPHMs was safer than western medicine. However, the increasing reports of adverse events associated with Chinese herbal medicines have excited attentions [32]–[34]. More and more TCM researchers began to investigate the specific mechanism of ingredient of Chinese herbs and its safety. TCM investigators for future study should be encouraged to monitor and report adverse events in clinical trials to GCP standards in order to evaluate the potential harms of CPHMs.

We have found that a systematic review titled ‘Chinese medicinal herbs for the common cold’ has been published in the Cochrane library in 2007 [35]. In this review, the author assessed the effect of Chinese medicinal herbs for the common cold. They only included RCT and included both CPHMs and individually prescribed herbal formulae. Our review is different from Zhang 2007 [35] not only in aim, but also in inclusion criteria, search strategy, analysis, and problem revealed. Our aim was to provide a well-round clinical evidence assessment of state authorized recommended CPHMs for common cold. Therefore we included all study designs and restricted CPHMs as those in the EDL.

The limitation of our review was that there were 6 studies that did not provided their diagnostic criteria, just mentioned that ‘common cold patients were included’. We did not exclude them with the intent to providing more information in our review. Thereafter, possibility existed that participants with other acute respiratory infections such as influenza were recruited.

In summary, a confirmative conclusion on the beneficial effect of CPHMs for common cold still cannot be drawn. To ensure evidence-based clinical practice, further rigorous placebo-controlled, randomized trials are warranted. Further TCM researchers should pay more attention to reducing risks of bias and other limitations of trials, and improving the reporting quality by complying with international reporting standards such as the CONSORT [36], in addition, more emphasis should be paid on the selection of control intervention and outcome measurement. Too much dependent on the presence of absence of fever is not recommended.

The rough results of this review have been published in abstract form in the Journal of Alternative and Complementary Medicine as a conference paper for the 2014 International Research Congress on Integrative Medicine and Health [37].

## Supporting Information

Table S1 **List of study design features.**
(DOCX)

Table S2 **Compositions and indications of 33 CPHMs listed in ‘China national essential drug list 2012’ for common cold.**
(DOCX)

Table S3 **List of the excluded studies with reasons.**
(DOCX)

Table S4 **The characteristics of included studies.**
(DOC)

Table S5 **Effect estimations of CPHMs for treatment of common cold in included trials.**
(DOCX)

Checklist S1 **PRISMA 2009 checklist.**
(DOC)

Appendix S1 **The scope and number of CPHMs in the ‘Chinese national essential drug list 2012’.**
(DOCX)
