# Supplementary material for: Chinese Proprietary Herbal Medicine Listed in ‘China National Essential Drug List’ for Common Cold: A Systematic Literature Review
Source: PLoS One. 2014 Oct 20;9(10):e110560. doi: 10.1371/journal.pone.0110560 (PMC4203808; doi:10.1371/journal.pone.0110560)
Supplement: Table S1 — List of study design features. (DOCX) [file pone.0110560.s001.docx]

Table S1. List of study design features

|  | **RCT** | **Q-RCT** | **NRCT** | **CBA** | **PCS** | **RCS** | **HCT** | **NCC** | **CC** | **XS** | **BA** | **CR/CS** |
| --- | --- | --- | --- | --- | --- | --- | --- | --- | --- | --- | --- | --- |
| *Was there a comparison:* |  |  |  |  |  |  |  |  |  |  |  |  |
| Between two or more groups of participants receiving different interventions? | Y | Y | Y | Y | Y | Y | Y | Y | Y | Y | N | N |
| Within the same group of participants over time? | P | P | N | Y | N | N | N | N | N | N | Y | N |
| *Were participants allocated to groups by:* |  |  |  |  |  |  |  |  |  |  |  |  |
| Concealed randomization? | Y | N | N | N | N | N | N | N | N | N | na | na |
| Quasi-randomization? | N | Y | N | N | N | N | N | N | N | N | na | na |
| By other action of researchers? | N | N | Y | P | N | N | N | N | N | N | na | na |
| Time differences? | N | N | N | N | N | N | Y | N | N | N | na | na |
| Location differences? | N | N | P | P | P | P | P | na | na | na | na | na |
| Treatment decisions? | N | N | N | P | P | P | N | N | N | P | na | na |
| Participants' preferences? | N | N | N | P | P | P | N | N | N | P | na | na |
| On the basis of outcome? | N | N | N | N | N | N | N | Y | Y | P | na | na |
| Some other process? (specify) |  |  |  |  |  |  |  |  |  |  |  |  |
| *Which parts of the study were prospective:* |  |  |  |  |  |  |  |  |  |  |  |  |
| Identification of participants? | Y | Y | Y | P | Y | N | P* | Y | N | N | P | P |
| Assessment of baseline and allocation to intervention? | Y | Y | Y | P | Y | N | P* | Y | N | N | na | na |
| Assessment of outcomes? | Y | Y | Y | P | Y | P | P | Y | N | N | P | P |
| Generation of hypotheses? | Y | Y | Y | Y | Y | Y | Y | Y | P | P | P | na |
| *On what variables was comparability between groups assessed:* |  |  |  |  |  |  |  |  |  |  |  |  |
| Potential confounders? | P | P | P | P | P | P | P | P | P | P | N | na |
| Baseline assessment of outcome variables? | P | P | P | Y | P | P | P | N | N | N | N | na |

**Abbreviations:** Y, Yes; P, Possibly; P*, Possible for one group only; N, No; na, not applicable; RCT, Randomized controlled trial; Q-RCT, Quasi-randomized controlled trial; NRCT, Non-randomized controlled trial; CBA, Controlled before-and-after study; PCS, Prospective cohort study; RCS, Retrospective cohort study; HCT, Historically controlled trial; NCC, Nested case-control study; CC,Case-control study; XS, Cross-sectional study; BA, Before-and-after comparison; CR/CS, Case report/Case series.
