# Supplementary material for: Chinese Proprietary Herbal Medicine Listed in ‘China National Essential Drug List’ for Common Cold: A Systematic Literature Review
Source: PLoS One. 2014 Oct 20;9(10):e110560. doi: 10.1371/journal.pone.0110560 (PMC4203808; doi:10.1371/journal.pone.0110560)
Supplement: Table S2 — Compositions and indications of 33 CPHMs listed in ‘China national essential drug list 2012’ for common cold. (DOCX) [file pone.0110560.s002.docx]

**Table S2.** Compositions and indications of 33 CPHMs listed in ‘China national essential drug list 2012’ for common cold

| **Name of CPHMs** | **Compositions** | **Indications** | **Dosage** |  |
| --- | --- | --- | --- | --- |
| Jiuwei Qianghuo pills | *Rhizoma seu Radix Notopterygii, Radix Saposhnikoviae, Rhizoma Atractylodis, Radix Angelica dahuricae, Radix Scutellaria baicalensis Georgi, Rhizoma Chuanxiong, Radix Rehmanniae, Radix Glycyrrhizae, Manchurian Wildginger* | For the external contraction wind-cold with damp, such as aversion to cold, fever, anhidrosis, headache, top-heaviness, body aching pain. | Taken with spring onions soup or warm boiled water, 6-9g each time, 2-3 times per day. |  |
| Jiuwei Qianghuo granules | *Rhizoma seu Radix Notopterygii, Radix Saposhnikoviae, Rhizoma Atractylodis, Manchurian Wildginger, Rhizoma Chuanxiong, Radix Angelica dahuricae, Radix Scutellaria baicalensis Georgi, Radix Rehmanniae, Radix Glycyrrhizae* | For the external contraction wind-cold with damp, such as aversion to cold, fever, anhidrosis, headache, top-heaviness, body aching pain. | Taken after mixing it with ginger decoction or boiled water, 15g each time, 2-3 times per day. |  |
| Ganmao Qingre granules | *Schizonepeta tenuisfolia Briq , Herba Mentha haplocalyx., Radix Saposhnikoviae , Radix Bupleuri , Folium Perillae , Radix Pueraria lobata (Willd) Ohwi., Radix Platycodonis , Semen Armeniacae Amarum , Radix Angelica dahuricae, Herba corydalis Bungeanae, Rhizoma Phragmites communis Trin* | For the wind-cold cold, headache, fever, aversion to cold, body pain, runny nose, cough and dry throat. | Taken after mixing it with boiled water, 12g per time, 2 times per day. |  |
| Ganmao Qingre capsules | *Schizonepeta tenuisfolia Briq , Herba Mentha haplocalyx., Radix Saposhnikoviae , Radix Bupleuri , Folium Perillae , Radix Pueraria lobata (Willd) Ohwi., Radix Platycodonis , Semen Armeniacae Amarum , Radix Angelica dahuricae, Herba corydalis Bungeanae, Rhizoma Phragmites communis Trin* | For the wind-cold cold, headache, fever, aversion to cold, body pain, runny nose, cough and dry throat. | Taken orally, 3 capsules (0.45g per capsule) per time, 2 times per day. | |
| Zhengchaihuyin granules | *Radix Bupleuri, Pericarpium Citri Reticulatae, Radix Saposhnikoviae, Radix Glycyrrhizae, Radix Paeoniae Rubra, Rhizoma Zingiberis Recens* | For the beginning of external contraction wind-cold, such as fever, aversion to cold, anhidrosis, headache, nasal congestion,sneezing, throat itching, cough, limbs aching pain. And the beginning of influenza, mild upper respiratory tract infection with above symptoms and signs. | Taken after mixing it with boiled water, 5g per time, 3times per day. | |
| Chaihu Injection | *Radix Bupleuri* | For the colds, influenza, malaria and other fever. | Intramuscular injection. 2-5ml per time, 1-2times per day. | |
| Yinqiao Jiedu pills | *Flos Lonicerae , Fructus Forsythiae, Herba Mentha haplocalyx., Schizonepeta tenuisfolia Briq, Semen Glycine Sojae, Fructus Arctii(Fry), Radix Platycodonis, Herba Loophatheri, Radix Glycyrrhizae* | For the wind-heat cold, fever, headache, cough, thrist, throat pain. | Taken orally with phragmites soup or warm boiled water, 6g per time, 2-3times per day. | |
| Yinqiao Jiedu granules | *Herba Mentha haplocalyx., Semen Glycine Sojae, Herba Loophatheri, Radix Glycyrrhizae, Flos Lonicerae, Schizonepeta tenuisfolia Briq, Radix Platycodonis, Fructus Forsythiae, Fructus Arctii* | For the wind-heat cold, such as fever, headache, cough, thrist, throat pain. | Taken after mixing it with boiled water, 15g per time, 3times per day. Sicker patient add one time per day. | |
| Yinqiao Jiedu capsules | *Flos Lonicerae, Fructus Forsythiae, Herba Mentha haplocalyx., Schizonepeta tenuisfolia Briq,Semen Glycine Sojae, Fructus Arctii（Fry）, Radix Platycodonis, Herba Loophatheri, Radix Glycyrrhizae* | For the wind-heat cold, such as fever, headache, cough, thirst, throat swelling and pain. | Taken orally, 4 capsules (0.4g per capsule) per time, 2-3 times per day. | |
| Yinqiao Jiedu soft capsules | *Flos Lonicerae, Fructus Forsythiae, Herba Mentha haplocalyx., Schizonepeta tenuisfolia Briq, Semen Glycine Sojae, Fructus Arctii(Fry), Radix Platycodonis, Herba Loophatheri, Radix Glycyrrhizae* | For the wind-heat cold, such as fever, headache, cough, thrist, throat pain; upper respiratory tract infection with above symptoms and signs. | Taken orally, 2 capsules (0.45g per capsule) per time, 3 times per day. | |
| Yinqiao Jiedu tablets | *Flos Lonicerae, Fructus Forsythiae, Herba Mentha haplocalyx., Semen Glycine Sojae, Schizonepeta tenuisfolia Briq, Fructus Arctii(Fry), Radix Platycodonis, Herba Loophatheri, Radix Glycyrrhizae* | For the wind-heat cold, such as fever, headache, cough, thrist, throat pain. | Taken orally, 4 tablets (0.55g per tablet) per time, 2-3 times per day. | |
| Xiongju Shangqing pills | *Radix Angelica dahuricae, Herba Mentha haplocalyx., Rhizoma Chuanxiong, Radix Saposhnikoviae, Radix Glycyrrhizae, Rhizoma Ligustici, Rhizoma Coptis chinensis Franch , Radix Scutellaria baicalensis Georgi, Schizonepeta tenuisfolia Briq, Radix Platycodonis, Flos Chrysanthemi, Fructus Forsythiae, Fructus Viticis, Rhizoma seu Radix Notopterygii, Fructus Gardenia jasminoides Ellis* | For the external contraction pathogenic wind, such as aversion to wind,fever, headache, nasal congestion, toothache. | Taken orally, 6g per time, 2 times per day. | |
| Xiongju Shangqing granules | *Rhizoma Chuanxiong, Flos Chrysanthemi, Radix Scutellaria baicalensis Georgi, Radix Angelica dahuricae, Radix Platycodonis, Fructus Gardenia jasminoides Ellis, Fructus Forsythiae, Radix Saposhnikoviae, Fructus Viticis(Fry), Schizonepeta tenuisfolia Briq, Rhizoma Coptis chinensis Franch , Radix Glycyrrhizae, Rhizoma seu Radix Notopterygii, Herba Mentha haplocalyx., Rhizoma Ligustici* | For the external contraction pathogenic wind, such as aversion to wind,fever, headache, nasal congestion, toothache. | Taken after mixing it with boiled water, 10g per time, 3 times per day. | |
| Xiongju Shangqing tablets | *Rhizoma Chuanxiong, Flos Chrysanthemi, Herba Mentha haplocalyx., Fructus Forsythiae, Fructus Viticis(Fry), Schizonepeta tenuisfolia Briq, Radix Saposhnikoviae, Radix Angelica dahuricae, Rhizoma seu Radix Notopterygii, Rhizoma Ligustici, Radix Platycodonis, Rhizoma Coptis chinensis Franch , Radix Scutellaria baicalensis Georgi, Fructus Gardenia jasminoides Ellis, Radix Glycyrrhizae* | For the external contraction pathogenic wind, such as aversion to wind, fever, headache, thin nasal discharge, toothache, laryngalgia. | Taken orally, 4 tablets (0.3g per tablet) per time, 2 times per day. | |
| Niuhuang Qinggan capsules | *Radix Scutellaria baicalensis Georgi, Flos Lonicerae, Fructus Forsythiae, Calculus Bovis Syntheticus, Concha Margaritifera Usta, Talcum powder* | For the cold induced by external contraction wind-heat, such as fever, cough, pharyngalgia. | Taken orally, 2-4 capsules (0.3g per capsule) per time, 3 times per day. For children, reduce the dosage with discretion or follow doctor's order. | |
| Xiaoer Baotaikang granules | *Fructus Forsythiae , Radix Rehmanniae , Bupleurum marginatum , Radix Scrophulariae , Folium Mori , Bulbus Fritillaria thunbergii Miq. , Herba Taraxaci , Baphicacanthus cusia (Nees) Bremek , Onosma , Radix Platycodonis , Semen Raphani , Radix Glycyrrhizae* | For children with external contraction wind-heat, such as fever, nasal discharge, cough | Taken after mixing it with warm boiled water, 3 times per day, under 1 year old, 2.6g per time; 1 to 3 years old, 4g per time; 3 to 12 years old, 8g per time. | |
| Zukamu granules | *Rhizoma Kaempferia galanga L., Flos Nymphaea Tetragona, Fructus Cordia dichotoma, Herba Mentha haplocalyx., Fructus Ziziphi Jujubae, Flos Matricaria recutita, Radix Glycyrrhizae, Fructus Althaea rosea （L.） Cavan., Radix et Rhizoma Rhei, Pericarpium Papaver somniferum L.* | For cold, cough, fever, anhidrosis, throat swelling and pain, nasal congestion and discharge. | Taken orally, 12g per time, 3 times per day. | |
| Xiaoer Resuqing oral liquid | *Radix Bupleuri , Radix Scutellaria baicalensis Georgi ,Radix Isatidis , Radix Pueraria lobata (Willd) Ohwi. , Flos Lonicerae , Cornu Bubali , Fructus Forsythiae , Radix et Rhizoma Rhei* | For children with cold induced by external contraction wind-heat, such as fever, headache, throat swelling and pain, nasal congestion and nasal discharge, cough, dry stool. | Taken orally, 3-4 times per day, under 1 year old, 2.5-5ml per time; 1 to 3 years old, 5-10ml per time; 3 to 7 years old, 10-15ml per time; 7 to 12 years old, 15-20ml per time. | |
| Xiaoer Resuqing granules | *Radix Isatidis, Radix Bupleuri, Radix et Rhizoma Rhei, Radix Pueraria lobata (Willd) Ohwi., Radix Scutellaria baicalensis Georgi, Flos Lonicerae, Fructus Forsythiae, Cornu Bubali* | For the wind-heat cold, fever, headache, red swollen throst, nasal congestion and yellow nasal discharge, cough, constipation. | Taken orally, 3-4 times per day, under 1 year old, 0.5-1g per time; 1 to 3 years old, 1-2g per time; 3 to 7 years old, 2-3g per time; 7 to 12 years old, 3-4g per time. | |
| Qingre Jiedu capsules | *Gypsum Fibrosum, Flos Lonicerae, Radix Scrophulariae, Radix Rehmanniae, Fructus Forsythiae, Fructus Gardenia jasminoides Ellis, Herba Gueldenstaedtia multiflora Bunge., Radix Scutellaria baicalensis Georgi, Radix Gentianae, Radix Isatidis, Rhizoma Anemarrhenae, Radix O.japonicus (L.f.) Ker-Gawl.* | For the treatment of influenza, upper respiratory tract infection. | Taken orally, 2-4 capsules (0.3g per capsule) per time, 3 times per day. | |
| Qingre Jiedu granules | *Rhizoma Coptis chinensis Franch , Cornu Bubali , Radix Scrophulariae , Flos Lonicerae , Radix Rehmanniae , Folium Isatidis , Fructus Forsythiae , Rhizoma Anemarrhenae ,Gypsum Fibrosum* | For the wind-heat cold. | Taken after mixing it with boiled water, 18g per time, 3 times per day. For children, reduce the dosage with discretion or follow doctor's order. | |
| Huoxiang Zhengqi liquid | *Rhizoma Atractylodis , Pericarpium Citri Reticulatae , Cortex Magnoliae officinalis(stir-bake with ginger juice) , Radix Angelica dahuricae, Poriae Cocos , Pericarpium Arecae , Rhizoma Pinelliae , Radix Glycyrrhizae extraction , Pogostemon cablin oil , Folium Perillae oil* | For the external contraction wind-cold, internal injury and damp stagnation, headache dizziness and top-heaviness, epigastric and abdominal distending pain, vomiting, diarrhea; gastrointestinal flu. | Taken orally, 5-10ml per time, 2 times per day, Shake well before using. | |
| Huoxiang Zhengqi oral liquid | *Rhizoma Atractylodis , Pericarpium Citri Reticulatae , Cortex Magnoliae officinalis(stir-bake with ginger juice) , Radix Angelica dahuricae , Poriae Cocos , Pericarpium Arecae , Rhizoma Pinelliae , Radix Glycyrrhizae extraction , Pogostemon cablin oil , Folium Perillae oil* | For the cold induced by external contraction wind-cold, internal injury and damp stagnation, or summer-damp, such as headache, dizziness and top-heaviness, stuffiness and oppression in chest and diaphragm, epigastric and abdominal distending pain, vomiting, diarrhea; gastrointestinal flu with above symptoms and signs. | Taken orally, 5-10ml per time, 2 times per day, Shake well before using. | |
| Huoxiang Zhengqi soft capsules | *Rhizoma Atractylodis , Pericarpium Citri Reticulatae , Cortex Magnoliae officinalis(stir-bake with ginger juice) , Radix Angelica dahuricae , Poriae Cocos , Pericarpium Arecae , Rhizoma Pinelliae , Radix Glycyrrhizae extraction , Pogostemon cablin oil , Folium Perillae oil* | For the cold induced by external contraction wind-cold, internal injury and damp stagnation, or summer-damp, such as headache, dizziness and top-heaviness, stuffiness and oppression in chest and diaphragm, epigastric and abdominal distending pain, vomiting, diarrhea; gastrointestinal flu with above symptoms and signs. | Taken orally, 2-4 capsules (0.45g per capsule) per time, 2 times per day. | |
| Shuanghuanglian mixture | *Flos Lonicerae, Radix Scutellaria baicalensis Georgi, Fructus Forsythiae* | For fever, cough, pharyngalgia induced by external contraction wind-heat. | Taken orally, 20ml per time, 3 times per day. | |
| Shuanghuanglian oral liquid | *Radix Scutellaria baicalensis Georgi , Flos Lonicerae , Fructus Forsythiae* | For the cold induced by external contraction wind-heat, such as fever, cough，pharyngalgia. | Taken orally, 20ml per time, 3 times per day. For children, reduce the dosage with discretion or follow doctor's order. | |
| Shuanghuanglian granules | *Flos Lonicerae, Radix Scutellaria baicalensis Georgi, Fructus Forsythiae* | For the cold induced by external contraction wind-heat, such as fever, cough，pharyngalgia. | Taken orally or take after mixing it with boiled water, 10g per time, 3 times per day; children under 6 months, 2.0-3.0g per day; 6 months to 1 year old, 3.0-4.0g per time; 1 to 3 years old, 4.0-5.0g per time; children over 3 years old, take with discretion. | |
| Shuanghuanglian capsules | *Flos Lonicerae, Radix Scutellaria baicalensis Georgi, Fructus Forsythiae* | For the wind-heat cold, such as fever, cough, pharyngalgia. | Taken orally, 4 capsules (0.4g per capsule) per time, 3 times per day. | |
| Shuanghuanglian tablets | *Radix Scutellaria baicalensis Georgi , Flos Lonicerae , Fructus Forsythiae* | For the cold induced by external contraction wind-heat, such as fever, cough，pharyngalgia. | Taken orally, 4 tablets (0.53g per tablet) per time, 3 times per day. For children, reduce the dosage with discretion or follow doctor's order. | |
| Yinhuang oral liquid | *Flos Lonicerae Extract, Radix Scutellaria baicalensis Georgi Extract* | For the external contraction wind-heat, the pattern of intense heat in lung and stomach, such as dry throat, pharyngalgia, laryngeal nuclear enlargement, thirst, fever; acute and chronic tonsillitis, acute and chronic pharyngitis, upper respiratory tract infection with above symptoms and signs. | Taken orally, 10-20ml per time, 3 times per day. For children, reduce the dosage with discretion. | |
| Yinhuang granules | *Flos Lonicerae, Radix Scutellaria baicalensis Georgi* | For the external contraction wind-heat, the pattern of intense heat in lung and stomach, such as dry throat, pharyngalgia, laryngeal nuclear enlargement, thirst, fever; acute and chronic tonsillitis, acute and chronic pharyngitis, upper respiratory tract infection with above symptoms and signs. | Taken after mixing it with boiled water, 2-4g per time, 2 times per day. | |
| Yinhuang capsules | *Flos Lonicerae Extract, Radix Scutellaria baicalensis Georgi Extract* | For acute and chronic tonsillitis, acute and chronic laryngopharyngitis, upper respiratory tract infection. | Taken orally, 2-4 capsules (0.3g per capsule) per time, 4 times per day. | |
| Yinhuang tablets | *Flos Lonicerae Extract, Radix Scutellaria baicalensis Georgi Extract* | For acute and chronic tonsillitis, acute and chronic laryngopharyngitis, upper respiratory tract infection. | Taken orally, 2-4 tablets (0.25g per tablet) per time, 4 times per day. | |

**Abbreviations:** CPM, Chinese patent medicine
