# Supplementary material for: Chinese Proprietary Herbal Medicine Listed in ‘China National Essential Drug List’ for Common Cold: A Systematic Literature Review
Source: PLoS One. 2014 Oct 20;9(10):e110560. doi: 10.1371/journal.pone.0110560 (PMC4203808; doi:10.1371/journal.pone.0110560)
Supplement: Table S3 — List of the excluded studies with reasons. (DOCX) [file pone.0110560.s003.docx]

**Table S3.** List of the excluded studies with reasons

|  | **Reference** | **Reason for exclusion** |
| --- | --- | --- |
| 1 | Chen ZH. (2006) Clinical observation of Zhengchaihu granule in treating exogenous fever. Shanghai Journal of Traditional Chinese Medicine 40:22-23. | Inappropriate control |
| 2 | Yin WP, He P, Xia J. (2008) Clinical observation of Xiaoer Baotaikang granules in treatment of 61 patients with wind-heat common cold. Yunnan Journal of Traditional Chinese Medicine and Materia Medica 29:35. | Inappropriate control |
| 3 | Yang XM. (2010) Clinical efficacy and safety study of Zukamu granules for treatment of common colds in children. China Modern Medicine 17:79. | Inappropriate control |
| 4 | Meng XJ. (2012) Clinical observation of Zukamu granules (Uighur medicine) for treatment of acute upper respiratory infection in children. Xinjiang Journal of Traditional Chinese Medicine 30:36-37. | Inappropriate control |
| 5 | Fang JY. (2011) Clinical observation of Xiaoer Resuqing granules combined with mint foot bathing in treatment of 65 patients with exogenous fever. Chinese Journal of Traditional Medical Science and Technology 18:432. | Inappropriate control |
| 6 | Wang H, Hu SY, Liu H. (2012) Multicenter-clinical trial of Xiaoer Resuqing granules in treating exogenous fever (wind-heat pattern) in children. Chinese Pediatrics of Integrated Traditional and Western Medicine 4:250-252. | Inappropriate control |
| 7 | Song Y, Sun YW. (2011) Efficacy and safety study of Xiaoer Resuqing granules for treatment of pediatric exogenous fever (wind-heat pattern). Heilongjiang Medicine Journal 24:549-551. | Inappropriate control |
| 8 | Wang SC, Luo HL. (2009) Clinical observation of Shufeng Jiedu capsule in treatment of 480 patients with upper respiratory infection. World Journal of Integrated Traditional and Western Medicine 4:872-875. | Inappropriate control |
| 9 | Liu GD, Qi HB. (1991) Efficacy observation of Huoxiang Zhengqi Liquid enema in treatment of summertime cold in 24 children patients. Hebei Journal of Traditional Chinese Medicine 13:12. | Inappropriate control |
| 10 | Chu L. (2007) Clinical control observation of Huoxiang Guizhi decoction combined with Huoxiang Zhengqi Liquid in treatment of common cold with dampness in 100 patients. Journal of Yunnan University of Traditional Chinese Medicine 30:45-53. | Inappropriate control |
| 11 | Lu ZQ. (2004) Efficacy observation of Shuanghuanglian oral liquid in treatment of 246 patients with common cold. Journal of Community Medicine 2:85-86. | Inappropriate control |
| 12 | Fen XQ, Lei Y. (2011) Efficacy observation of Shuanghuanglian granules for treatment of upper respiratory infection in children patients. Modern Journal of Integrated Traditional Chinese and Western Medicine 20:2129-2130. | Inappropriate control |
| 13 | Zou JW, Song Y. (2011) Efficacy and safety study of Shuanghuanglian table for treatment of common cold (wind-heat pattern). Heilongjiang Medicine Journal 24:543-546. | Inappropriate control |
| 14 | Hu GL, Wu CS. (2008) Randomized controlled study of Shuanghuanglian table for treatment of common cold (wind-heat pattern). Chinese Archives of Traditional Chinese Medicine 26:891-892. | Inappropriate control |
| 15 | Hu K, Jiang Y, Shi MJ, Hu CH, Liu ZL, et al. (2008) 102 cases of acute respiratory infection treated with Lianhua Qingwen capsule. Herald of Medicine 27:1337-1340. | Inappropriate control |
| 16 | Hu GF. (2011) Evaluation of the effect of Lianhua Qingwen capsule in treatment of acute upper respiratory tract infection. Practical Journal of Cardiac Cerebral Pneumal and Vascular Disease 19:832-833. | Inappropriate control |
| 17 | Wang YB, Xie YL, Yang YM, Xia L, Liu LM, et al. (2008) The efficacy and safety of Lianhua Qingwen capsule on viral cold. Conference papers of the 4th international conference on collateral disease :374-376 | Inappropriate control |
| 18 | Xun HJ, Du MX. (2011) Efficacy observation of Lianhua Qingwen capsule in treatment of viral cold. Guide of China Medicine 9:306-307. | Inappropriate control |
| 19 | Yin WP, He P. (2008) Clinical study of Xiaoer Baotaikang granules in treatment of wind-heat common cold. Conference papers of the 25th national symposium on higher education in pediatric medicine :350-352. | Duplication publication |
| 20 | Chen Q. (2010) Efficacy observation and nursing of Chaihu injection of Quchi ( LI 11 ) acupoint injection in treating pediatric exogenous fever. Nei Mongol Journal of Traditional Chinese Medicine 29:177. | Plagiarized publication |
| 21 | Gao QF. (1999) Shuanghuanglian oral liquid in treatment of 60 patients with pediatric upper respiratory tract infection. Chinese Journal of Primary Medicine and Pharmacy 6:378-379. | Not commom cold |
| 22 | Pan Y. (1997) Huoxiang Zhengqi Liquid in treatment of common cold with fever and diarrhea in 16 children patients. Jilin Journal of Traditional Chinese Medicine 17:18. | Not commom cold |
| 23 | Liang BY. (2004) Clinical observation of Yupingfeng for prophylaxis and treatment of common cold in children. Jilin Journal of Traditional Chinese Medicine 24:21. | Not commom cold |
| 24 | Song NP, Hu NP, Zhu S, Hna Q, Guo DZ. (1991) Clinical efficacy observation of Xiaoer Resuqing oral liquid in treatment of 96 patients with pediatric upper respiratory tract infection and fever. Henan Traditional Chinese Medicine 11:12-13. | Not commom cold |
| 25 | Ding Y, Wang YX. (1992) Clinical efficacy observation of Xiaoer Resuqing oral liquid in treatment of 148 patients with pediatric exogenous fever. China Journal of Traditional Chinese Medicine and Pharmacy 7:36-38. | Not commom cold |
| 26 | Pa L, Ru KY. (2000) Clinical observation of Zukamu granules for treatment of 50 patients with upper respiratory infection. Chinese Journal of Ethnomedicine and Ethnopharmacy :205-206. (volume not available) | Not commom cold |
| 27 | Sun B, He HZ. (2011) Lianhua Qingwen capsule in treatment of viral cold: a systematic review. Conference papers of the 7th international congress of collateral disease :326-329. | Not commom cold |
| 28 | Yang YM. (2000) Shuanghuanglian oral liquid in treatment of influenza in 96 children patients. Occupation and Health 16:95. | Influenza |
| 29 | Zhang RY, Wang L. (2011) Clinical observation of combine traditional Chinese and western medicine in treatment of influenza in 40 patients. Practical Clinical Journal of Integrated Traditional Chinese and Western Medicine 11:23-24. | Influenza |
| 30 | ARKEN M, Mi NWE. (2009) Safety evaluation of Zukamu granules for treatment of common cold and fever in children patients. Xinjiang Journal of Traditional Chinese Medicine 27:40-41. | No outcome measurement |
