# Supplementary material for: Chinese Proprietary Herbal Medicine Listed in ‘China National Essential Drug List’ for Common Cold: A Systematic Literature Review
Source: PLoS One. 2014 Oct 20;9(10):e110560. doi: 10.1371/journal.pone.0110560 (PMC4203808; doi:10.1371/journal.pone.0110560)
Supplement: Table S4 — The characteristics of included studies. (DOC) [file pone.0110560.s004.doc]

**Table S4. The characteristics of included studies**

| **Name of CPHMs** | **Pharmacological action** | **Administration** | **Study ID** | **Design** | **TCM pattern differentiation** | **Participants** | **Outcomes** |
| --- | --- | --- | --- | --- | --- | --- | --- |
| Chaihu injection | Antipyretic, anti-inflammatory effects and enhance immunity | Acupuncture injection in ear (acupuncture point not reported) | Dai 1995 | CS | Not reported | N:84;  M/F: 46/38;  Age: 1-14 years | Duration of fever |
|  |  | Retention enema | Li 1997 | CS | Not reported | N=30;  M/F: 21/9;  Age: 18 under 12 years, and 12 exceed 12 years. | Defervescence rate within 4 hours |
|  |  | Retention enema | Wang 2002 | CS | Not reported | N=26;  M/F: 15/11;  Age: 8 months-4 years | Defervescence rate within 3 hours |
|  |  | Nasal dripping | Zhang 2009 | CS | Not reported | N=20;  M/F: not reported.  Age: 3 under 5 years, 10 between 20 to 69 years, and 5 exceed 70 years. | Defervescence rate within 5 hours |
|  |  | Not reported | Wang 1999 | CS | Not reported | N=60;  M/F: 38/22;  Age: 5-45 years. | Clinical symptoms improvement rate within 3 days |
| Chaihu injection | Antipyretic, anti-inflammatory effects and enhance immunity | Acupuncture point injection (LI 11) in T1 and intramuscular injection in T2 | Lv 2010 | RCT | Not reported | N=253 (100 in T1, 52 in T2, 50 in C1, and 51 in C2);  M/F: not reported;  Age: not reported; | Duration of fever and defervescence rate within 3 days |
| Qingre Jiedu granules | Not reported | Oral intake | Xu 2010 | CS | Wind-heat type of common cold | N=90;  M/F: 34/56;  Age: 15-40 years | Clinical symptoms improvement rate within 3 days |
|  |  |  | Zhao 2012 | RCT | Wind-cold type of common cold | N=192 (106/86);  M/F: 64/42 in T and 54/32 in C;  Age: 3-14 years | Body temperature and clinical symptoms improvement rate within 73 days |
| Huoxiang Zhengqi liquid | Antiemetic, analgesic, antispasmodic effects and enhance immunity of cells | Oral intake | Jiang 2012 | CS | Wind-cold, endogenous damp stagnation, or summer-heat and dampness type of common cold | N=1560;  M/F: 791/769;  Age: 5-83 years (mean 41.5 years) | Clinical symptoms improvement rate (time did not report) |
| Ganmao Qingre granules | Antiviral, antibacterial, antipyretic and anti-inflammatory effects | Oral intake | Di 2012 | RCT | Wind-cold type of common cold | N=60 (30/30);  M/F: 18/12 in T and 16/14 in C;  Age: 6 months-12 years | Clinical symptoms improvement rate within 3 days |
| Shuanghuanglian oral liquid | Antiviral, antibacterial, ant-iinflammatory and antipyretic effects | Oral intake | Wang 1997 | RCT | Not reported | N=100 (50/50);  M/F: not reported;  Age: 6 months-14 years | Clinical symptoms improvement rate within 3 days |
| Xiaoer Baotaikang granules | Antipyretic, antibacterial, antiviral effects and enhance immunity | Oral intake | Wu 2012 | RCT | Wind-heat type of common cold | N=100 (50/50);  M/F: not reported;  Age: 4.28±3.37 years in T, and 4.56±3.44 years in C. | Clinical symptoms improvement rate within 3 days |
| Xiaoer Resuqing oral liquid | Antipyretic, antibacterial, antiviral, antitussive and expectorant effects | Oral intake | Li 2007 | RCT | Not reported | N=120 (65/55);  M/F: 75/45;  Age: 8 months-7 years | Clinical symptoms improvement rate within 5 days |

**Abbreviations:** CPM, Chinese patent medicine;
