# Supplementary material for: Chinese Proprietary Herbal Medicine Listed in ‘China National Essential Drug List’ for Common Cold: A Systematic Literature Review
Source: PLoS One. 2014 Oct 20;9(10):e110560. doi: 10.1371/journal.pone.0110560 (PMC4203808; doi:10.1371/journal.pone.0110560)
Supplement: Table S5 — Effect estimations of CPHMs for treatment of common cold in included trials. (DOCX) [file pone.0110560.s005.docx]

**Table S5.** Effect estimations of CPHMs for treatment of common cold in included trials

| **Name of CPHMs** | **Comparison** | **Outcomes** | **Effect estimate (95%CI)** |
| --- | --- | --- | --- |
| Chaihu injection | Saline | Duration of fever (hours) | MD* -33.32 (-35.71 to -30.93) |
|  |  | Defervescence rate within 3 days | RR* 14 (1.96 to 99.94) |
|  | Ribavirin | Duration of fever (hours) | MD -0.99 (-6.02 to 4.04) |
|  |  | Defervescence rate within 3 days | RR 0.78 (0.22 to 2.76) |
| Qingre Jiedu granules | Moroxydine hydrochloride and Oseltamivir | Body temperature after 3 days | MD 0.06 (0.01 to 0.11) |
|  |  | Clinical symptoms improvement rate with 3 days | RR* 1.38 (1.16 to 1.65) |
| Ganmao Qingre granules | Pediatric paracetamol | Clinical symptoms improvement rate within 5 days | RR 1.31 (0.78 to 2.19) |
| Shuanghuanglian oral liquid | Ribavirin | Clinical symptoms improvement rate within 3 days | RR* 4.00 (2.26 to 7.08) |
| Xiaoer Baotaikang granules | Amoxicillin | Clinical symptoms improvement rate within 3 days | RR* 2.67 (1.56 to 4.55) |
| Xiaoer Resuqing oral liquid | Moroxydine hydrochloride | Clinical symptoms improvement rate within 5 days | RR* 1.43 (1.15 to 1.77) |

## Abbreviations: CI, confidence interval; RR, risk ratio; MD, mean difference; *, results favors CPMs.
