# Supplementary material for: Chinese Proprietary Herbal Medicine Listed in ‘China National Essential Drug List’ for Common Cold: A Systematic Literature Review
Source: PLoS One. 2014 Oct 20;9(10):e110560. doi: 10.1371/journal.pone.0110560 (PMC4203808; doi:10.1371/journal.pone.0110560)
Supplement: Appendix S1 — The scope and number of CPHMs in the ‘Chinese national essential drug list 2012’. (DOCX) [file pone.0110560.s007.docx]

**Appendix 1.** The scope and number of CPHMs in the ‘Chinese national essential drug list 2012’

| **Ⅰ. Internal Medicine (137)** | |
| --- | --- |
| **1. Superficies-releasing prescription (12)** | 1.1 Release superficies with pungent-warm (3) |
|  | 1.2 Release superficies with pungent-cool (7) |
|  | 1.3 Release both exterior and interior (1) |
|  | 1.4 Reinforce healthy *qi* and release superficies (1) |
| **2. Purgative prescription (1)** | 2.1 Moisten Intestines and relaxing bowels (1) |
| **3. Heat-clearing prescription (18)** | 3.1 Clear heat and purge fire (4) |
|  | 3.2 Clear heat and relieve toxicity (4) |
|  | 3.3 Clear and dispel summer-heat (3) |
|  | 3.4 Clearing heat from internal organs (7) |
| **4. Interior-warming prescription (7)** | 4.1 Warm the energizer and disperse cold (4) |
|  | 4.2 Replenish *qi* and restore pulse (3) |
| **5. Phlegm-resolving, cough-stopping and dyspnea-relieving prescription (14)** | 5.1 Warm and resolve cold-phlegm (2) |
|  | 5.2 Clear and resolve heat-phlegm (3) |
|  | 5.3 Moisten lung and resolve phlegm (4) |
|  | 5.4 Disperse accumulations and resolve phlegm (1) |
|  | 5.5 Disperse wind and clear heat (1) |
|  | 5.6 Invigorate spleen and antitussive (1) |
|  | 5.7 Antasthmatic drug (2) |
| **6. Resuscitative prescription (5)** | 6.1 Clear heat for resuscitation (3) |
|  | 6.2 Resolve phlegm for resuscitation (2) |
| **7. Healthy-reinforce prescription (19)** | 7.1 Invigorate spleen and replenish *qi* (4) |
|  | 7.2 Invigorate spleen and harmonious stomach (2) |
|  | 7.3 Invigorate spleen and nourish blood (2) |
|  | 7.4 Nourish yin and tonify kidney (1) |
|  | 7.5 Nourishing yin and falling fire (1) |
|  | 7.6 Nourish kidney and liver (2) |
|  | 7.7 Warmly invigorating kidney yang (3) |
|  | 7.8 Tonifying *qi*-blood (1) |
|  | 7.9 Supplementing qi and nourishing yin (3) |
| **8. Tranquilizing prescription (3)** | 8.1 Nourish heart and induce tranquilization (3) |
| **9. Hemostatic prescription (1)** | 9.1 Cool blood and stop bleeding (1) |
| **10. Stasis-dispelling prescription (23)** | 10.1 Activate blood and dispel stasis (5) |
|  | 10.2 Replenish *qi* and activate blood (6) |
|  | 10.3 Resolve stasis and soothe chest (2) |
|  | 10.4 Resolve stasis and unblock pulse (4) |
|  | 10.5 Regulate *qi* and activate blood (4) |
|  | 10.6 Nourish yin and activate blood (1) |
|  | 10.7 Dispel stasis and relieve toxicity (1) |
| **11. *Qi*-regulating prescription (8)** | 11.1 Soothe liver and relieve depression (3) |
|  | 11.2 Soothe liver and harmonize stomach (5) |
| **12. Digestive and evacuative prescription (3)** | 12.1 Promote digestion and remove food stagnation (3) |
| **13. Wind-relieving prescription (10)** | 13.1 Course and dissipate external wind (1) |
|  | 13.2 Pacify liver and extinguish wind (2) |
|  | 13.3 Dispel wind and resolve phlegm (1) |
|  | 13.4 Nourish blood and dispel wind (3) |
|  | 13.5 Dispel wind and unblock collateral (3) |
| **14. Dampness-dispelling prescription (11)** | 14.1 Dispel cold and eliminate dampness (2) |
|  | 14.2 Alleviate edema and induce dieresis (3) |
|  | 14.3 Clear heat and relieve stranguria (2) |
|  | 14.4 Resolve stasis and relieve stranguria (1) |
|  | 14.5 Reinforce healthy *qi* and dispel dampness (2) |
|  | 14.6 Replenish kidney and relieve stranguria (1) |
| **15. Lipid-regulating prescription (1)** | 15.1 Resolve turbidity and reduce lipid (1) |
| **16. Astringent prescription (1)** | 16.1 Tonify kidney and reduce urination (1) |
|  |  |
| **Ⅱ. Surgical Medicine (11)** | |
| **1. Heat-clearing prescription (8)** | 1.1 Clear heat and remove dampness (1) |
|  | 1.2 Clear heat and relieve toxicity (4) |
|  | 1.3 Relieve stranguria and alleviate stones (1) |
|  | 1.4 Clear heat and alleviate edema (1) |
|  | 1.5 Soften hardness and dissipate nodulation (1) |
| **2. Meridian-warming, *q*i-regulating and blood-activating prescription (1)** | 2.1 Dissipate nodulation and alleviate edema (1) |
| **3. Blood-activating and stasis-resolving prescription (2)** | 3.1 Resolve stasis and unblock pulse (1) |
|  | 3.2 Alleviate edema and activate blood (1) |
|  |  |
| **Ⅲ. Gynecological Medicine (20)** | |
| **1. Blood-regulating prescription (5)** | 1.1 Activate blood and resolve stasis (2) |
|  | 1.2 Resolve stasis and stop bleeding (1) |
|  | 1.3 Astringent and stop bleeding (1) |
|  | 1.4 Nourish blood and soothe liver (1) |
| **2. Heat-clearing prescription (6)** | 2.1 Clear heat and remove dampness (3) |
|  | 2.2 Clear heat and relieve toxicity (2) |
|  | 2.3 Promote flow of *qi* and break stasis (1) |
| **3. Healthy-reinforce prescription (5)** | 3.1 Nourish blood and regulate *qi* (1) |
|  | 3.2 Replenish *qi* and nourish blood (2) |
|  | 3.3 Nourish yin and induce tranquilization (2) |
| **4. Nodulation-dissipating prescription (4)** | 4.1 Alleviate edema and dissipate nodulation (1) |
|  | 4.2 Activate blood and resolve stasis (3) |
|  |  |
| **Ⅳ. Ophthalmological Medicine (7)** | |
| **1. Heat-clearing prescription (4)** | 1.1 Clear heat and dissipate wind (2) |
|  | 1.2 Purge fire and improve vision (2) |
| **2. Healthy-reinforce prescription (3)** | 2.1 Nourish yin and liver (2) |
|  | 2.2 Replenish *qi* and nourish yin (1) |
|  |  |
| **Ⅴ. Otorhinolaryngological Medicine (13)** | |
| **1. Otopathy (2)** | 1.1 Nourish kidney and pacify liver (2) |
| **2. Rhinopathy (5)** | 2.1 Ventilate lung and open orifice (1) |
|  | 2.2 Clear heat and open orifice (1) |
|  | 2.3 Disperse wind and clear heat (2) |
|  | 2.4 Reinforce healthy qi and release superficies (1) |
| **3. Throat disease and stomatosis (6)** | 3.1 Resolve phlegm and relieve sore throat (2) |
|  | 3.2 Nourish yin and clear heat (2) |
|  | 3.3 Clear heat and cool blood (1) |
|  | 3.4 Clear heat and relieve toxicity (1) |
|  |  |
| **Ⅵ. Orthopedics and traumatological Medicine (15)** | |
|  | 1.1 Reuniting bone, muscle and ligament (2) |
|  | 1.2 Activate blood and resolve stasis (4) |
|  | 1.3 Activate blood and unblock collateral (3) |
|  | 1.4 Dispel wind and activate collateral (5) |
|  | 1.5 Tonify kidney and strengthen bones (1) |
